# 

**Published:** 1896-07

**Authors:** 


### TABLE OF CONTENTS

ORIGINAL COMMUNICATIONS. PAGE

Empyema of the Antrum of Highmore. By FREDERIC C. COBB, M.D., Boston, Mass. . . . . . . . . . . . . . . . . . . . . . . . . . 405

The Principles and Applications of Cataphoresis. By WENDELL C. PHILLIPS, M.D., New York . . . . . . . . . . . . . . . . . . . . . . . . . 418

Cocoaine. By SAMUEL A. HOPKINS, M.D., Boston, Mass. . . . . . . . . 431

Loretin, the New Antiseptic. By S. ELDRED GILBERT, D.D.S., Philadelphia . . . . . . . . . . . . . . . . . . . . . . . . . . . . 441

A Roentgen Ray Converter for Internal Use. By WILLIAM ROLLINS, Boston, Mass. . . . . . . . . . . . . . . . . . . . . . . . . . . . 445

REPORTS OF SOCIETY MEETINGS.

American Academy of Dental Science . . . . . . . . . . . . . . . . . . 446

The New York Institute of Stomatology . . . . . . . . . . . . . . . . . 461

EDITORIAL.

The Vacation Period . . . . . . . . . . . . . . . . . . . . . . . . . 472

The American Dental Association . . . . . . . . . . . . . . . . . . . 475

BIBLIOGRAPHY . . . . . . . . . . . . . . . . . . . . . . . . . . . 476

OBITUARY.

Dr. W. E. Magill . . . . . . . . . . . . . . . . . . . . . . . . . . 478

CURRENT NEWS.

National Association of Dental Examiners . . . . . . . . . . . . . . . 479

New Jersey State Dental Society . . . . . . . . . . . . . . . . . . . 479

Illinois State Dental Society . . . . . . . . . . . . . . . . . . . . 479

Cincinnati Academy of Dentistry . . . . . . . . . . . . . . . . . . . 480

SELECTIONS.

Remedy for Laceration in Extraction . . . . . . . . . . . . . . . . . . 480

